# Retraction: A new compound of thiophenylated pyridazinone IMB5043 showing potent antitumor efficacy through ATM-Chk2 pathway

**DOI:** 10.1371/journal.pone.0347912

**Published:** 2026-04-27

**Authors:** The PLOS One Editors

After publication of this article [1], concerns were raised regarding Figs 3, 5, and S1. Specifically, the following panels appear similar to each other:

- S1 Fig phospho-ATR in [1] and P-IκBα lanes 1-4 in [2]
- S1 Fig ATR in [1] and p65 lanes 1-4 in Fig 3A in [2].
- Fig 3 phosph-p53 in [1] and Fig 5C cleaved caspase-3 in [1]
- Fig 3 Phospho-ATM and P-STAT3 HCT-116 lanes 2-5 in Fig 4C in a later article by a different author group [4] when flipped.

First author JG stated that the Fig 3 p-ATM panel is incorrect, which invalidates key findings of the article [1] as this panel does not correspond to the SMMC-7721 cells treated with IMB5043 as described, and that therefore, this error invalidates the key data supporting the article’s core conclusion regarding IMB5043 activating the ATM-Chk2 pathway.

Regarding the above concerns with S1 Fig, the first author stated that these were due to inadvertent use of the same raw image file for two different experimental conditions, but they were unable to clarify whether these panels are correct as the images underlying S1 Fig are no longer available. The first author also stated that the Fig 3 phosph-p53 panel is incorrect and the Fig 5C cleaved caspase-3 panel is correct, but that the underlying blots for Fig 5C are no longer available.

During post-publication discussions, original western blot images were provided for Fig 3; however, these do not appear to match the corresponding panels in [1].

In addition, during post-publication reassessment of this article, the following cell lines reported in this study have been identified as either contaminated or misclassified:

- The SMMC7721 cell line reported as a human hepatocellular carcinoma cell line in this study [1] has been identified as a contaminated cell line that was previously shown to be a HeLa derivative instead [5,6].
- The HepG2 cell line reported as a human hepatocellular carcinoma cell line in this study [1] has been identified as a misclassified cell line that was previously shown to originate from a hepatoblastoma instead [7].

In light of the above concerns, which call into question the integrity and reliability of the results in [1], the *PLOS One* Editors retract this article.

JG did not respond to the final editorial decision. YZheng, YW, WS, YL, XL, SS, RS, and YZhen either did not respond directly or could not be reached.

After publication of this article [1], it was also raised that Fig 5A 1µM in [1] appears similar to Fig 5D NC in a later article by a different author group [3].
